# Relationship Study of The Verified Human Epidermal Growth Factor Receptor 2 Amplification with Other Tumor Markers and Clinicohistopathological Characteristics in Patients with Invasive Breast Cancer, Using Chromogenic In Situ Hybridization 

**DOI:** 10.22074/cellj.2019.6219

**Published:** 2019-06-15

**Authors:** Abdolazim Sarli, Hossein Mozdarani, Nasser Rakhshani, Sohail Mozdarani

**Affiliations:** 1 Department of Medical Genetics, Faculty of Medical Sciences, Tarbiat Modares University, Tehran, Iran; 2 Firoozgar Hospital, Gastrointestinal and Liver Diseases Research Center, Iran University of Medical Sciences, Tehran, Iran; 3 Cytogenome Medical Genetics Laboratory, Chamran Medical Building, Ale Ahmad Highway, Tehran, Iran

**Keywords:** Breast Cancer, Chromogenic In Situ Hybridization, HER-2, Tumor Markers

## Abstract

**Objective:**

Human epidermal growth factor receptor 2 (*HER-2*), as a crucial factor involved in about 20% of breast cancer cases, is one of the most reliable tumor markers to determine prognosis and therapeutic trend of this disease. This marker is generally assessed by immunohistochemistry (IHC) technique. In the cases that result of IHC test cast doubt (+2), the test should be repeated or validated by applying in situ hybridization techniques, like chromogenic in situ hybridization (CISH). In this regard, the goal of current study was to figure out the link between different clinicopathological characteristics of patients suffering from invasive breast cancer, using tumor markers, hormone receptor (HR) and HER-2. Comparing IHC and CISH techniques for evaluating diagnostic value and usefulness of HER-2 were also the other objective of this study.

**Materials and Methods:**

Based on this retrospective study, histological markers of 113 individuals suffering from invasive breast cancer -such as estrogen receptor (ER), progesterone receptor, *HER-2* receptor, E-cadherin, CK5/6, vimentin and Ki67 were examined by IHC technique. HER-2 amplification of all patients was also evaluated by CISH. Clinicopathological information of the patients was also extracted from medical documents and their associations with tumor markers were statistically evaluated.

**Results:**

There is a significant relationship between tumor size, CK5/6 and tumor grade with HR status. Similar relationship was observed between* HER-2* status and HR status, as well as vascular invasion (P<0.05). The comparison of HER-2 amplification showed no complete concordance of the result obtained from these two techniques, with score +3.

**Conclusion:**

Since the status of *HER-2* is very important in decision making of the treatment process, CISH technique is recommended in the malignant conditions as the primary test, instead of IHC. In this study, we also determined that HER-2 expression is greatly correlated with ER- and PR- status. This might propose a better prognosis for *HER-2^+^* patients.

## Introduction

Breast carcinoma is a multifactorial ailment comprised of noticeable biological subtypes with vast variation in clinical, pathological and molecular features having various prognostic and therapeutic implications. The nature of this malignancy is interconnected with its clinical outcomes (1). It is important to note that up to 21 distinct histological subtypes and at least four various molecular subtypes of breast cancer, correlating with distinct risk factors, have thus far been diagnosed which are biologically different in presentations and results (2, 3).

Evaluating different biological markers -including presence or absence of hormone (i.e. estrogen or progesterone) receptors (named respectively HR^+^ or HR^-^) and excessive level of human epidermal growth factor receptor 2 (*HER- 2*)- is the most applicable method for identifying the subtype of the cancer (4), leading to classification of some distinct subtypes of breast cancer: luminal A (HR+/*HER-2^-^*), triple negative (HR^-^/*HER-2^-^*), luminal B (HR^+^/*HER-2^+^*) and HER- 2-enriched (HR^-^/*HER-2^+^*) tumors (5).

*HER-2* gene product is a 185-kDa trans-membrane growth factor receptor with tyrosine kinase activity involved in cellular signaling. It is responsible for regulating cell growth and development (6). Clinical studies show that *HER-2* gene is amplified in 20-30% of all breast cancers (7), out of which overexpression is the direct result of this gene amplification in ~90-95% of cases (6). This phenomenon is a remarkable prognosis factor associated with lymph node metastasis, HR- tumors, high-grade tumor, great recurrence risk after operation, weak response to common chemotherapy and no chance of long-term survival (8).

*HER-2* expression is an important factor in therapeutic decision-making of breast cancer, since HER-2 protein (*HER- 2* gene product) is targeted for specific treatment by humanized recombinant monoclonal antibody Trastuzumab. So that, this drug could only be applied for treatment of patients with *HER-2^+^* malignancy (9).

These days, expression of estrogen receptor (ER), progesterone receptor (PR) and HER-2 are measured by immunohistochemistry (IHC) technique, as a prognostic factor applied in the routine protocol of breast cancer treatment. In this technique, amplification of HER-2 is reported in three scores: i. No amplification of the targeted gene which is considered as +1, ii. An interface that does not indicate whether there is any increase in the HER-2 protein level and it is shown as +2, in addition to iii. The definite amplification of HER-2 which is considered as +3. The patient’s IHC scored +2 should be rechecked by IHC or evaluated by some in situ hybridization techniques, like fluorescent in situ hybridization (FISH) or chromogenic in situ hybridization (CISH). Some studies implicate that CISH is more sensitive than IHC (10).

In this study, we examined sensitivity of the results obtained from IHC and CISH tests. For this purpose, HR (ER and PR) and HER-2 proteins of breast cancer patients were evaluated by these two techniques. In addition, all demographic and histopathological characteristics of the patients were recorded. The results of IHC test were scored as +1, +2 and +3, and compared to CISH test representing status of HER-2 expression (HER-2+ and HER-2- groups). Finally, histopathological characteristics and tumor subtypes obtaining from these two techniques were analyzed to detect meaningful correlations.

## Materials and Methods

This retrospective study was conducted over a period of four years at Mehr Hospital Pathology Department (Tehran, Iran). Over this time, 113 mastectomy specimens were obtained. In all cases, clinical features and tumor studies, including ER, PR, E-cadherin, CK5/6, vimentin and Ki67, as well as HER-2, were performed on formalin-fixed paraffinembedded (*FFPE*) tissue samples. Disease of specimens was completely gross based on a standard protocol. In addition, other data including tumor size, side of the breast, invasive ductal or lobular carcinoma, in situ component, grade and tumor vascular invasion were recorded.

Tissue was subjected to routine processing and sections were stained with hematoxylin and eosin stain (11). The histopathological criteria were diagnosed based on WHO classification and the samples were graded, applying Modified Blooms Richardson Grading System. In addition, antibodies were applied to ER, PR, *HER-2* receptor, E-cadherin, CK5/6, vimentin and Ki67.

### Evaluation of progesterone receptor, estrogen receptor and HER-2 using IHC

Slices were made in thicknesses of 3-4 micrometers and placed on polyethylene lysine-coated slides. They were next deparaffined in xylene followed by distilling off with ethanol. Paraffin and healing slices were next set in 3% hydrogen peroxide solution (Sigma-Aldrich, USA). Antigenic reagents were performed by a 0.01 M citrate buffer solution with pH=6 for 20 minutes in microwave. In the next step, the sections were separately incubated with 7 antibodies (all from AbCam, UK) for 60 minutes at 37˚C: Monoclonal Mouse Anti-ErbB2 Affibody® Molecule, Monoclonal Mouse Antihuman Estrogen, Monoclonal Mouse Antihuman Progesterone, Monoclonal Mouse Anti-E Cadherin antibody, Monoclonal Mouse Anti-Cytokeratin 5+6 antibody (D5/16 B4), Monoclonal Mouse Anti-vimentin antibody and Monoclonal Mouse Anti-Ki67 antibody. Normal tissue surrounding the tumor was used as the control of HER- 2, ER and PR. We could also quantify ER, PR staining by utilizing Allred score. All the slides were quantified by giving proportional scores regarding the percentage of cells, nuclear stain presence and intensity score considering the intensity of staining. The proportional score (PS) is as follows: 1% of cells representing nuclear stain, 10% of cells demonstrating nuclear stain, 33% of cells showing nuclear stain, 66% of cells expressing nuclear stain, 100% of cells showing nuclear stain. Intensity score (IS) is as follows: 0-negative weak staining, 1- intermediate staining and 2- strong staining. Total score (TS) is considered as follow: sum of PS+intensity. TS greater than 2 is regarded positive for significant expression of ER and PR. Immunohistochemical assessment of HER- 2 overexpression was considered positive, considering more than 10% of cells is severely stained (+3 score). In ambiguous cases (+2 score), they had to be confirmed by CISH.

### Chromogenic in situ hybridization

In this experiment, paraffin blocks were divided into 5-6 micron sections (at least 2 sections) to evaluate expression of *HER-2* marker. We also categorized all original breast tumor tissues with either modified radical mastectomy or breastconserving surgery to confirm diagnosis of the invasive carcinomas.

The test has been conducted by applying CISH, based on Zyto Dot: 2C SPEC HER-2/CEN-17 dual Probes Kit protocol (Zytovision, Germany). The PD-12 probe contains digoxigenin-labeled polynucleotides targeting sequences of the *HER-2* gene and DNA-labeled polynucleotides targeting alpha-satellites of the centromere of chromosome 17 causing formation of green and red signals, illustrated by light microscopy (×40 objective lens). All of these reactions were performed in two days, following four steps, in line with the kit protocol (www.zytovision.com).

**Fig.1:**
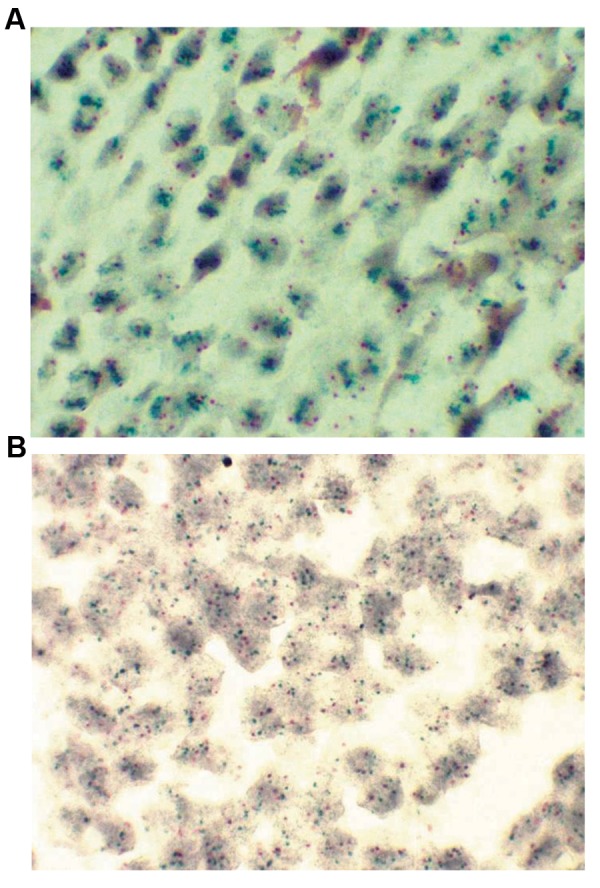
Illustration of a CISH result performed on the patient tumor sample. **A.** HER-2+ sample, whereby green-to-red ratio is more than 2 and **B.** HER-2- sample, whereby green-to-red ratio is less than 2 (red color is the indicator of chromosome 17 centromeric probes, and green color is the indicator of specified probes of HER-2 gene) (scale bar: 100 μm).

CISH hybridization signal of one single copy of *HER-2* gene, appears like a distinct dark green dot-shaped signal, while the signal of one single copy of chromosome 17 centromeric region appears as a distinct bright red dotshaped signal which can clearly be distinguished from the background counterstained with hematoxylin (Fig .1). All slides were analyzed and the results were recorded and scored in accordance to the American Society of Clinical Oncology/ College of American Pathologists (ASCO/CAP) guidelines. Briefly, the numbers of CEN-17 and *HER-2* signals were counted in 100 non-overlapping invasive cancer cell nuclei, applying at least three distinct tumor fields (when possible). *HER-2* signal heterogeneity was not regarded in this study. Where the mean *HER-2*/CEN-17 ratio in any field is 2 or greater, the tumor is, therefore, amplified. Where the ratio is less than 2 whereas average of HER-2 signal number per cell is equal to or less than 2, it is not amplified. Cases with a ratio of less than 2 and *HER-2* signal number per cell between 4 and 6 were considered as equivocal borderline results and after counting an additional 20 nuclei according to new ASCO/CAP guideline 2018 version (12, 13), final decision on the degree of amplification was made.

### Ethical considerations

All experiments were performed in accordance with relevant guidelines and regulations. All FFPE samples were obtained from the Mehr Hospital. This study was approved by the Ethics Committee of Tarbiat Modares University (registered number: 52D/4922), Tehran, Iran. Written informed consent was obtained from each participant before FFPE sample collection.

### Statistical analysis

In this study, chi-square data analysis and Fisher’s exact test were applied. All statistical analyses were conducted using Statistics Package for Social Sciences (SPSS) version 18 at the significant level of P<0.05. Quantitative variables were reported as mean ± SD and qualitative variables were also reported as frequency (%).

## Results

In the present study, various clinicopathological parameters in 113 cases of infiltrating ductal (102 cases) and lobular (11 cases) carcinoma were analyzed and summarized in Table 1. The range of breast cancer patient age onset was between 27 and 95 years old. Demographic data is given in Table 1.

The ER and PR relationship with pathological and demographic features, as well as clinical characteristics of patients are presented in Table 2.

**Table 1:** Demographic characteristics of ductal carcinoma breast cancer patients


Characteristics		Number of subjects (%)

Age (Y)		
	Mean	54.05 ± 12.729
	Range	27-95
Stage at diagnosis		
	Stage I	37 (32.7)
	Stage II	28 (24.7)
	Stage III	27 (23.8)
Not determined		21 (18.5)
Breast involvement		
	Right breast	67 (59.3)
	Left breast	42 (37.5)
	Bilateral involvement	4 (3.2)
Size of tumor		
	More than 2 cm	81 (71)
	Less than 2 cm	32 (29)
Grade of tumor		
	Grade 1	25 (22.1)
	Grade 2	57 (50.4)
	Grade 3	22 (19.4)
Not determined		9 (7.9)
Vascular invasion		57 (51.8)
Type of breast cancer		
	Ductal carcinoma	101 (90.2)
	Lobular carcinoma	9 (8.0)
	In situ component of tumor	57 (50.9)
Hormone receptor status (IHC)		
	ER^+^	85 (75.9)
	ER^-^	27 (24.1)
	PR^+^	69 (61.6)
	PR^-^	43 (38.4)
	ER^-^ and PR^-^	30 (27)
HER-2		
	+1 (negative)	27 (24.1)
	+2 (equivocal)	65 (58.0)
	+3 (positive)	21 (17.9)
Biomarkers		
	E-cadherin positive	33 (68.8)
	CK5/6 positive	9 (14.1)
	Vimentin positive	4 (7.3)
	Ki67	92 (95.8)
HER-2 (CISH)		
	Amplified	35 (31.3)
	Not amplified	77 (68.8)
	Triple negative	18 (16.1)


PR; Progesterone receptor and ER; Estrogen receptor.

**Table 2:** Comparison of biomarker, demographic and clinical variables in terms of different combinations of ER and PR


Variable		ER^-^/PR^+^ or ER^+^/PR^-^	ER^+^/PR^+^	ER^-^/PR^-^	P value (Chi-square test)

Age					
	≤45	7 (41.2)	17 (26.2)	5 (16.7)	0.18
	>45	10 (58.8)	48 (73.8)	25 (83.3)	
Tumor size					
	≤2	13 (76.5)	26 (40.0)	7 (23.3)	0.002
	>2	4 (23.5)	39 (60.0)	23 (76.7)	
Breast					
	Right	12 (70.6)	36 (55.4)	19 (63.3)	0.52
	Left	4 (23.5)	28 (43.1)	10 (33.3)	
	Bilateral	1 (5.9)	1 (1.5)	1 (3.3)	
Invasive ductal carcinoma					
	No	1 (5.9)	7 (10.8)	3 (10.0)	0.91
	Yes	16 (94.1)	58 (89.2)	27 (90)	
Invasive lobular carcinoma					
	No	17 (100.0)	58 (89.2)	28 (100.0)	0.38
	Yes	0 (0.0)	7 (10.8)	0 (0.0)	
In situ component					
	No	7 (41.2)	30 (46.2)	18 (60.0)	0.35
	Yes	10 (58.8)	35 (53.8)	12 (40.0)	
Grade					
	1	5 (31.3)	18 (28.6)	2 (8.0)	0.01
	2	10 (62.5)	35 (55.6)	12 (48.0)	
	2	1 (6.3)	10 (15.9)	11 (44.0)	
Vascular invasion					
	Negative	7 (41.2)	34 (52.3)	12 (42.9)	0.57
	Positive	10 (58.8)	31 (47.7)	16 (57.1)	
Stage					
	I	2 (66.7)	11 (55.0)	4 (44.4)	0.73
	II	0 (0.0)	6 (30.0)	2 (22.2)	
	III	1 (33.3)	3 (15.0)	3 (33.3)	
E-cadherin					
	Negative	3 (42.9)	10 (35.7)	2 (15.4)	0.38
	Positive	4 (57.1)	18 (64.3)	11 (84.6)	
CK5/6					
	Negative	1 (10.0)	2 (5.9)	14 (70.0)	0.04
	Positive	9 (90.0)	32 (94.1)	6 (30.0)	
Vimentin					
	Negative	6 (100.0)	33 (97.1)	12 (80)	0.11
	Positive	0 (0.0)	1 (2.9)	3 (20.0)	
Ki67					
	Negative	0 (0.0)	3 (5.3)	1 (4.0)	0.83
	Positive	13 (100)	54 (94.7)	24 (96.0)	


PR; Progesterone receptor and ER; Estrogen receptor. Data are presented as n (%).

According to Table 2, only association of CK5/6 with different combinations of ER and PR results is statistically noticeable (P<0.05). There is no significant association of E-cadherin, vimentin and Ki67 clinical variables with different combinations of ER and PR results (P>0.05). Chi-square analyses also indicate no significant association of tumor size and grade variables with different combinations of ER and PR (P<0.05). On the other hand, one of the goals of this study was to investigate potential association of HER-2 status (positive or negative result) using CISH technique with pathological and clinical variables of the patients. Results of this objective are reported in Tables 3 and 4.

**Table 3:** Comparison of histological variables in patients with positive and negative CISH HER-2 result


Variable	CISH HER-2		P value(Chi-square test)
	Positive	Negative	

ER Positive Negative	20 (57.1)15 (42.9)	59 (76.6)18 (23.4)	0.03
PR Positive Negative	16 (45.7)16 (54.3)	52 (67.5)25 (32.5)	0.02
E-cadherin Positive Negative	11 (78.6)3 (21.4)	22 (64.7)12 (35.3)	0.34
**CK5/6 Positive Negative**	2 (10.0)18 (90.0)	7 (15.9)37 (84.1)	0.70
**Vimentin Positive Negative**	0 (0.0)18 (100.0)	4 (10.8)33 (89.2)	0.14
**Ki67 Positive Negative**	29 (96.7)1 (3.3)	62 (95.4)3 (4.6)	0.14


Data are presented as n (%).

**Table 4:** Comparison of demographic and clinical variables in patients with positive and negative CISH HER-2 result


Variable		*CISH HER-2*		P value(Chi-square test)
		Positive	Negative	

Age				
	≤45	8 (20.0)	22 (28.6)	0.33
	>45	28 (80.0)	55 (71.4)	
Tumor size				
	≤2	13 (37.1)	33 (42.9)	0.56
	>2	22 (62.9)	44 (57.1)	
Breast				
	Right	25 (71.4)	42 (54.5)	0.21
	Left	9 (25.7)	33 (42.9)	
	Bilateral	1 (2.9)	2 (2.6)	
Invasive ductal carcinoma				
	Yes	34 (97.1)	67 (87.0)	0.09
Invasive lobular carcinoma				
	Yes	1 (2.9)	8 (13.0)	0.17
In situ component				
	Positive	18 (51.4)	39 (50.6)	0.93
	Negative	17 (48.6)	38 (49.4)	
Grade				
	1	5 (14.7)	20 (28.6)	0.30
	2	21 (61.8)	36 (51.4)	
	3	8 (23.5)	14 (20.0)	
Vascular invasion				
	Positive	25 (71.4)	32 (42.7)	0.005
	Negative	10 (28.6)	43 (57.3)	
Stage				
	I	6 (40.0)	11 (64.7)	0.08
	II	3 (20.0)	5 (29.4)	
	III	6 (40.0)	1 (5.9)	


Data are presented as n (%).

**Fig.2:**
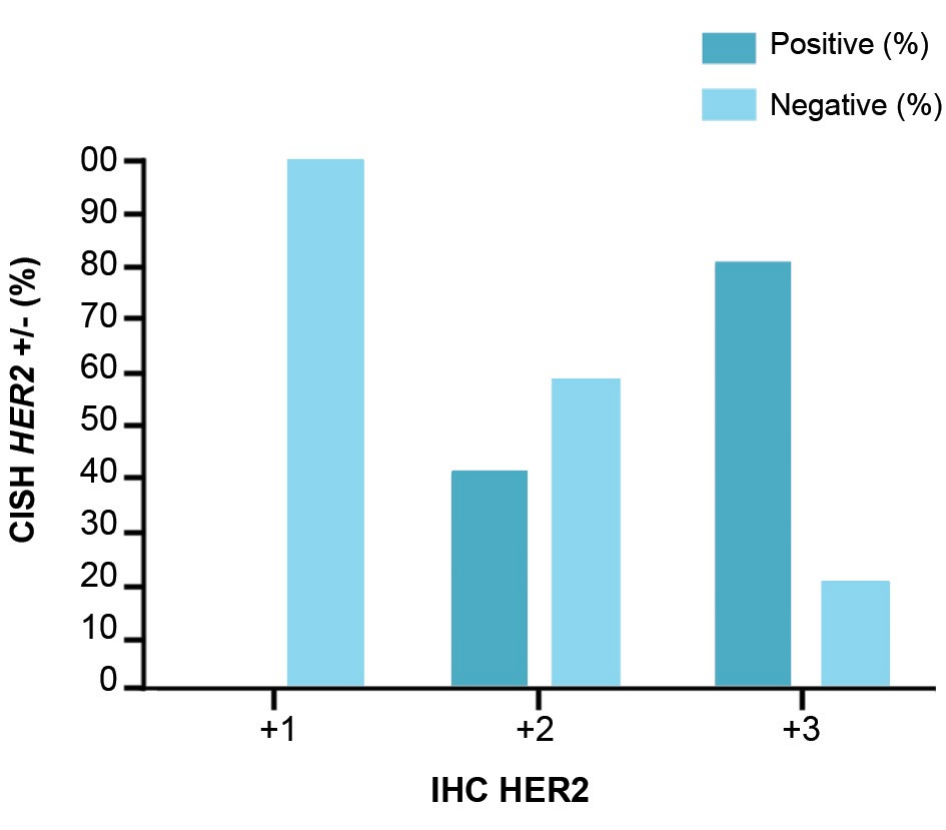
Comparison of two techniques, CISH and IHC, in HER-2 amplification detection. HER2 status is defined according to IHC in 3 states: +1 (HER2 negative) where the cell membranes are not stained or less than 10% of the cells are stained. +2 (Equivocal), in which the membrane contains more than 10% of the cells weakly or moderately stained and +3 (HER2 positive) in which the membrane of more than 10% of the cells is completely and severely stained.

According to Table 4, results obtained from chi-square analysis revealed that only association of vascular invasion with CISH HER-2 status is statistically significant (P<0.05). Finally, in order to detect HER-2 amplification, sensitivity and specificity of CISH were compared to IHC technique. The results are illustrated in Figure 2.

As shown in Figure 2, there are differences in *HER- 2* amplification frequency of +2 and +3 scores between CISH and IHC methods. In the cases of +1 score (i.e. *HER-2* negative) using IHC, the results were confirmed by CISH technique. But, in the cases of +2 and +3 scores using IHC (i.e. *HER-2* positive), CISH technique reveals contradictory cases.

## Discussion

Breast cancer is the main cause of around 9-34% of all patient malignancies in women, and about 1 million new cases are recognized annually around the world (14). In addition, breast cancer is a widespread type of malignancy occurring in women living in developed countries and it is the fifth cause of death among all cancers (14, 15). One of the important issues in diagnosis and treatment of breast cancer is impossibility of early detection (16). Therefore, improving diagnostic process will have a remarkable output in the consequences of breast cancer.

Prognostic and diagnostic factors play important role in several aspects, including perception of the disease process in patients, predicting disease outcome, choosing the right treatment and planning for implementation of the extra treatment process. ER and PR status determination is very important in choosing the right treatment of breast cancer (17). These receptors are also considered as prognostic factors during hormone therapy (18).

In our study, the onset age mean of patients is 54 which, to some extent, is higher than the patients in other studies. In a study performed by Erbil et al. (19) age mean of the total number of 231 patients was 45 years and in the other study carried out by Mohaghegh et al. (20), the reported average of age was 48.3 years. In a study conducted by Payandeh et al. (21), the age mean of patients was 46.39 years.

In our study, 85 (75.9%) of the total cases were ER+, while 69 (61.6%) of them were PR+. Therefore, in our population study, ER+ patients have considerable prevalence and in comparison with other studies, the number of ER+ individuals is greater than that of PR+ (22, 23).

There are controversial reports on association of HR with clinicopathological features of the patients. In our study, no significant association between ER and PR with pathological features of E-cadherin, vimentin and Ki67 was observed. Furthermore, although there was a correlation between tumor size and grade of the disease, in addition to CK5/6, we did not notice a remarkable link between HR status and clinical features as well as demographic information including age, stage of disease, invasive lobular and in situ component. Thike et al. (24) showed that there is no association between age and HR status. However, in another study performed by Jalava et al. (23) an association between age and ER status was determined. Jalava et al. (23) and Aaltomaa et al. (25) showed no specific relationship between tumor size and HR status. However, in this study, we determined that size of the tumor in HR+ patients was more than 2 cm. Moreover, Moreover, in a study a lack of correlation has been reported between HR and histological analysis of carcinoma cells, while in several studies a correlation between HR+ and invasive lobular cancer was reported. HR+ status is generally common in patients with low tumor stage according to the result obtained from our study. However, due to the lack of samples with diagnosed stage of disease, it was not statistically significant. Our results also indicate that HR^+^ tumors have more +2 score than HR- tumors. This indication is in line with several, but not all, studies (23). The basal type cytokeratin CK4/5 expression correlates with poor prognostic features, such as early recurrence, axillary lymph node positivity, high tumor grade, Ki-67 positivity and ER negativity (25). Our results showed that CK4/5 is often seen in HR- samples, in accordance with those of Choccalingam et al. (26) reports who also demonstrated that basal-like breast cancer expression, defined by basal cytokeratin expression, correlates with negative hormonal status and shorter disease-free intervals. Trastuzumab drug is used to treat patients suffering from *HER-2^+^* invasive breast cancer tumors. In *HER-2^-^* cases, however, administration of this drug not only fails to have any benefit for the patients, but also it results in cardiotoxicity and additional costs for patients (27).

In the present study, 35 (31%) patients showed overexpression of *HER-2*. The worldwide prevalence of women with *HER-2^+^* breast cancer is 15-20% of the total affected cases which is also related to invasive forms of the disease (12). *HER-2^+^* cancer cells can produce two millions copy of the relevant protein on their surfaces which is almost 100 fold more than normal cells. This promotes the cancer cells to grow and reproduce faster. An essential step in the signaling pathway leading to cancer cell growth is the dimerization of the *HER-2* receptor protein (28). Several studies have reported the relationship between *HER-2* and prognostic factors (29). In a research study, Konecny et al. (30) showed a reverse relationship of HER-2 with ER and PR status. Additionally, in a cohort study, a reverse correlation of *HER-2* with HR status as well as a positive correlation between tumor grade and overexpression of *HER-2* was reported (31).

In our study, most of the *HER-2^+^* patients were aged more than 45 years old. According to our results, HER-2 showed a significant relationship with tumor vascular invasion; in most of the *HER-2^+^* patients, tumor also had vascular invasion, while in the case of HER-2- patients, vascular invasion showed no statistical difference. Other prognostic factors related to breast cancer showed no statistical relationship with HER-2 status. In this study, HER-2 gene expression significantly associated with ER- and PR- status. This is similar to the study of Ariga et al. (32). It has been recommended that this association could reflect a better prognosis. However, the other studies revealed that ER+/*HER-2^+^* status accompanied with a poorer survival rate than ER+/*HER-2^-^* status. Therefore, it sounds that *HER-2* expression is a better predicator of response to hormonal therapy than ER status itself.

Whereas these results are in accordance with previous studies, more sample size and clinicopathological information is needed to reach more precise and comprehensive results. In this way, individuals who are candidates for *HER-2* examination, in the process of treatment could be diagnosed at the early stage of disease using CISH technique, with no need of IHC technique application.

In this study, we analyzed the frequency of patient sample features by IHC and CISH methods. As mentioned previously, the results of 18 (16.1%) patients, analyzed by IHC and CISH techniques, were triple negative and 30 (27%) patients were ER- and PR- synchronously. However, this finding contradict with the previously reported frequency of triple negative breast cancer patients was 54.83% among infiltrating ductal carcinoma. In addition, Sandhu et al. (33) in another study reported 31% prevalence of triple negative breast cancer in 7223 of Indian patients.

As mentioned before, we used CISH technique in our study. In *HER-2* examination, one of the remarkable privileges of CISH over IHC is the increase of specificity and sensitivity. The other advantage of in situ hybridization method for *HER-2* is that this examination be done through a comparative way with a reference sequence in one reaction on a slide which results in reduction of errors and increase of accuracy. Relatively qualitative method is another limitation of IHC technique, leading to inaccuracy of +1, +2 and +3 scores distinction related to HER-2. Moreover, this method is affected by technical errors, especially experience of operator (34).

In the case of solid tumors, CISH is better and the relative slides could be conserved longer, compared to FISH method. Additionally, detection of gene amplification is more beneficial using CISH in contrast to FISH, regarding that: i. In permanent staining, samples can be archived, ii. Bright field microscopy application would be feasible, iii. Identification of the target cells is easy, and iv. Tumor heterogeneity is easily assessed (35). In this study, we compared the results of CISH with IHC tests by examining a number of breast cancers.

Herceptin is an antibody-based drug utilized to treat breast cancer, by targeting overexpression of HER-2 protein, as it is observed in about one-third of breast cancer patients. Therefore, Herceptin is prescribed for HER-2+ patients. On the other hand, prescribing this medication for patients who are not diagnosed with conclusive HER- 2 gene expression may lead to adverse side-effects and even faster disease progression as well as economically imposing high costs to the patients’ family and public health system. Usually, +1 score is considered as nonamplification of HER-2 in IHC tests.

Currently, IHC tests are performed on most patients with breast cancer referring to laboratories in order to test for ER, PR, E-cadherin, CK5/6, vimentin and Ki67, among which *HER-2* gene amplification is examined to prescribe and use Herceptin. In IHC technique for HER-2 is classified to +1, +2, and +3 scores. While the +1 score is considered as *HER-2* non-amplified class, the +3 score is considered as definitely amplified *HER-2*. The +2 score is considered as equivocal, meaning that there is uncertainties in the *HER-2* expression of patients. Therefore, either IHC tests should be repeated or the sample evaluation should be validated by FISH or CISH test (10), imposing more costs and time consequently. As previously mentioned, definitive answer to the HER- 2 status is crucial for making decision to prescribe Herceptin.

In this study, we also compared the results of *HER-2* amplifications by IHC and CISH techniques. According to results, CISH technique is considered more reliable than IHC. This comparisons show that only the cases with +1 score is considered non-amplified in IHC, fully validated by CISH method. Interestingly, the +2 score, which are considered equivocal results, account for 58% (65 patients) of all cases. In other words, only less than half of the patients receive the ultimate result using this test and their results must be verified by repeating experiment or utilizing other techniques such as CISH or FISH. Therefore, despite cheaper cost of IHC technique, it seems that would be a more rational to perform CISH test in patients from the beginning. It is worthy to note that *HER-2^+^* patients with +2 score results (74% of the cases) were verified through CISH method.

The results obtained from CISH test showed 2 patients, out of 20 HER-2+ cases with +3 IHC score, were actually HER-2-. False positivity of these 2 patients, as a significant IHC problem to test HER-2 protein overexpression, might lead to wrong process of their disease treatment. A minority of cases of breast cancer scoring *HER-2* (+3) by IHC using Herceptin test may not be associated with findings obtained from CISH, which confirms that the CISH technique has a higher accuracy and sensitivity (36).

In this project, we also calculated the rate of similarity between IHC and CISH results from two aspects: i. Proportion of the negative (+1) or positive (+3) cases obtained from IHC, to CISH and ii. Proportion of the cases identified as +2 IHC, to the CISH HER-2+. Considering all these results, the rate of similarity between IHC and CISH in the cases of +1 and +3 scores was around 95.8% (45/47) and the concordance between +2 score and positive cases of CISH were around 26.2% (17/65). This result may be due to polysemy of chromosome 17 in breast tumors which may lead +2 IHC score of the cases to show false positive (37, 38). Totally, the overall concordance of these two techniques for detecting HER-2+ tumors is about 61%, while in the other studies, this concordance was varied from 52 to 82% (39). In other studies, the relationship between results of FISH/CISH techniques and IHC techniques has been reported. For instance, in a study performed by Bahreini et al. (40), it was demonstrated that 36% of +2 IHC score cases, identified by FISH technique, were positive and 64% were negative.

## Conclusion

Since the results of *HER-2* status is important for making decision of the treatment process, CISH technique is recommended to test *HER-2* expression in the malignant and invasive conditions rather than IHC. Additionally, in the presented study, *HER-2* expression was significantly linked to ER- and PR- status that may reflect a better prognosis.
